# Supplementation of polyunsaturated fatty acids (PUFAs) and aerobic exercise improve functioning, morphology, and redox balance in prostate obese rats

**DOI:** 10.1038/s41598-021-85337-9

**Published:** 2021-03-18

**Authors:** Allice Santos Cruz Veras, Rayana Loch Gomes, Maria Eduarda Almeida Tavares, Inês Cristina Giometti, Ana Paula Mattoso Miskulin Cardoso, Beatriz da Costa Aguiar Alves, Sabrina Alves Lenquiste, Luiz Carlos Marques Vanderlei, Giovana Rampazzo Teixeira

**Affiliations:** 1 Postgraduate Program in Movement Sciences, São Paulo State University (UNESP), Presidente Prudente, SP Brazil; 2 Department of Physical Education, School of Technology and Sciences, São Paulo State University (UNESP), 19060-900, Street Roberto Simonsen, Presidente Prudente, SP 305 Brazil; 3 Department of Physiotherapy, São Paulo State University (UNESP), Presidente Prudente, SP Brazil; 4 Faculty of Nutrition, University of Oeste Paulista (UNOESTE), Presidente Prudente, SP Brazil; 5 Multicentre Graduate Program in Physiological Sciences, São Paulo State University (UNESP), Araçatuba, SP Brazil; 6 Faculty of Veterinary Medicine, Universidade Do Oeste Paulista (UNOESTE), Presidente Prudente, SP Brazil; 7 Faculty of Medicine of ABC (FMABC), Santo André, São Paulo, Brazil; 8 Experimental Laboratory of Exercise Biology (LEBioEx), São Paulo State University (UNESP), Presidente Prudente, SP Brazil

**Keywords:** Cell biology, Molecular biology, Oncology

## Abstract

The high-fat diet (HFD) stimulates an increase in lipids and can be prejudicial for harmful to prostatic morphogenesis. Polyunsaturated fatty acid (PUFAs) have anti-inflammatory and antioxidant action in some types of cancer. The combination of aerobic physical exercise and PUFA can be more effective and reduce the risk of death. The study evaluates the effects of aerobic physical exercise associated with omega-3 (fish and chia oils), on the ventral prostate of Wistar rats those fed with HFD. Here, we report that HFD modified the final body weight and the weight gain, decreased the expression of the androgen receptor and increased prostatic inflammation via TNF-α produced damage prostatic like intraepithelial neoplasia. The supplementation with fish oil decreases final body weight, reduced BCL-2 and inflammation compared to chia oil; aerobic physical exercise associated with fish oil reduced lipids circulant and prostatic, increased proteins pro-apoptotic expression and reduced IL-6 (p < 0.0001) and TNF-α potentiating the CAT (p = 0.03) and SOD-1 (p = 0.001) expression. Additionally, the chia oil increased the NRF-2 (p < 0.0001) and GSS (p = 0.4) genes. PUFAs reduced the damage caused by excessive high-fat diet in the prostate so that there is greater effectiveness in omega-3 intake, it is necessary to associate with aerobic physical exercise.

## Introduction

The prostate is an accessory gland that secretes several nutrients that make up the seminal fluid, essential for the nutrition and motility of sperm. Prostate alterations affect men frequently, it is estimated by National Cancer Institute^1^ that approximately 65,840 new cases of prostate cancer occurred in Brazil in 2020, equivalent to 29.2% of the population. According to American Cancer Society (ACS), researchers estimate that in the US in 2021, almost 1.9 million new cancer cases will be diagnosed, and more than 600,000 people will die from cancer. Systemic metabolic alterations associated with increased consumption of saturated fat and obesity are linked with increased risk of prostate cancer progression and mortality, but the molecular underpinnings of this association are poorly understood^1^.

The omega-3 PUFAs is a metabolite that has anti-inflammatory properties^2^ are extensively investigated throughout the body and shown a low correlation with cardiovascular diseases in obesity^3^ and which may be effective in either prevention or prostate cancer treatment^4^. The omega-3 fatty acids in fish include eicosapentaenoic acid and docosahexaenoic acid (300 mg/g/oil)^5^ and chia seeds contains the highest proportion of alpha-linolenic acid (ALA, 0.6 g/g/oil) from any known vegetable source^6^. When analyzing the effects of omega-3 PUFAs on cell proliferation and survival^7^, these fatty acids exhibit anti-inflammatory properties through their impact on prostaglandin synthesis and eicosapentaenoic acid (EPA) and docosahexaenoic acid DHA have inhibitory effects on prostate cancer growth and progression^8^. Additionally, a wide range of mechanisms by which omega-3 fatty acids affect cancer development have been clarified. Additionally, is possible that activation of EPA-induced gamma peroxisome proliferation receptors (PPAR-γ), which can interfere with the translocation of factor nuclear kappa B (NF-κB) to the nucleus, reducing associated cytokines, tumor necrosis factor (TNF-α) and interleukin-6 (IL-6). Notably, pro‑inflammatory cytokines such as interleukin (IL)-1, IL-6 and TNF are able to affect cancer risk. The mechanism antitumor activity of omega*-*3 PUFAs is tightly linked to their ability to trigger autophagy and apoptosis, reducing expression of BCL-2 and stimulation of the BAX and BAD mitochondrial and set the stage for an effective treatment of tumors possessing functional p53; however, since p53 is frequently mutated in human cancers^8^.

In the prostate, the panorama of alterations caused by the fish or chia oil supplementation, rich diets, EPA and DHA or just ALA, is controversial and there is much to be related to the high-fat diet. Fatty acids are the primary energy source for prostate cancer cells and androgens upregulate fatty acid synthase (FASN), the enzyme responsible for the de novo synthesis of fatty acids which is linked to an increase in prostatic adenocarcinoma^9^. Sterol response element binding protein-1 (SREBP-1) is a positive regulator of FASN expression through binding elements in the FASN promoter and it is possible that diets rich in omega-3 PUFAs inhibit the cleavage of SREBP-1 and consequent downregulation of FASN. Additionally, was demonstrated that SREBP-1 regulated AR promoter activity and expression, and cell viability in prostate^10^. Furthermore, SREBP-1 increased reactive oxygen species (ROS) levels via increased NADPH oxidase 5 (Nox5) expression in prostate cancer cells. ROS has been shown to induce signal transduction, survival and progression of cancer cells^11^. We trust that omega-3 PUFAs supplementation can regulate lipogenesis and ROS signaling by increasing the production of antioxidant defenses and regulation of AR in the prostate, however it is not clear in the literature about the best proportion of EPA, DHA and ALA and their potentials effects.

Aerobic physical exercise regulates body energy expenditure helping to decrease body fat^12^, predominantly using fatty acid oxidation (AG) as an energy source^13^, regulating the profile and metabolism of lipids and glucose^14^, and reducing plasma lipid levels^15^, chronic inflammation^16^ and antioxidant enzymes^17^. Although the most optimal intensity, volume, and modality of exercise to combat disease have yet to be established in the literature, there are reports that moderate physical exercise increases apoptosis in prostate cancer cells^18^. Different types of exercises can modulate the negative effects of poor lifestyle, obesity, smoking and in the obesogenic environment, thus, It has already been demonstrated that physical training modulates positively the prostate of rats submitted to high-fat diet^19^. Therefore, we investigated in the present study the ability of fish and chia oil supplementation associated with aerobic exercise to improve metabolic changes and inhibit prostate diseases associated with a high-fat diet.

## Results

### Aerobic physical exercise is associated with PUFA supplementation and effects on body and adiposity

As expected, the high-fat diet increased weight gain, when compared to the CT group; and the supplementation of fish and chia oil, alone or associated with physical exercise significantly reduces the weight gain when compared to the HF group (Table 1). Adipose reserves differed significantly across groups as shown by differences in epididymal fat (p = 0.004; Table 1), mesenteric fat (p = 0.0008; Table 1), retroperitoneal fat (p = 0.002; Table 1), and fat index (p = 0.0001; Table 1). Post-hoc analysis revealed that the HF group had significantly greater epididymal fat, mesenteric, and retroperitoneal fat, and a greater fat index compared to the CT and exercise groups (Table 1). Adipose tissue and fat index in animals subjected to aerobic training and HF were comparable to the CT group. Though non-significant, retroperitoneal fat and fat index were lower in animals following aerobic physical training with fish oil and chia oil supplementation compared with the HF + FO and HF + CO groups (Table 1). The absolute prostate weight and relative prostate weights were reduced in the HF, HF + CO, and HF + FO groups, but no significant differences across groups (Table 1).

**Table 1:** Data on initial and final body weight, weight gain, absolute and relative prostate weights, absolute and relative fats, epididimal. mesenteric and retroperitoneal adipose tissues of trained animals, fish and chia oil intake, submitted to high-fat diet for 14 weeks.

Variables	CT	HF	HF + Ex	HF + FO	HF + FO + Ex	HF + CO	HF + CO + Ex
Initial body weight (g)	209.80 ± 5.22	199.80 ± 9.29	194.10 ± 8.25	191.70 ± 7.80	193.70 ± 8.40	195.60 ± 6.34	198.8 ± 5.57
Final body weight (g)	409.60 ± 7.27	410.30 ± 6.26	395.20 ± 8.89	382.50 ± 9.16	372.20 ± 9.21	380.10 ± 12.2	374.60 ± 8.413
Weight gain (g)	189.90 ± 6.69	224.00 ± 4.21	201.10 ± 3.71	190.80 ± 6.94	175.10 ± 5.42	188.10 ± 9.24	182.20 ± 9.15
Absolute prostate weight (g)	0.19 ± 0.01	0.15 ± 0.009	0.18 ± 0.01	0.16 ± 0.01	0.18 ± 0.02	0.14 ± 0.01	0.17 ± 0.01
Relative prostate weight (g)	0.04 ± 0.005	0.03 ± 0.004	0.04 ± 0.005	0.03 ± 0.005	0.04 ± 0.007	0.03 ± 0.005	0.04 ± 0.004
Total fat (g)	18.77 ± 1.09	28.08 ± 1.66^b^	22.42 ± 2.12	23.16 ± 1.87	19.66 ± 1.30	22.75 ± 2.54	19.24 ± 1.47
Relative fat (%)	4.56 ± 0.18	7.08 ± 0.28^ac^	5.18 ± 0.33	6.42 ± 0.25^ae^	4.98 ± 0.27^b^	5.91 ± 0.46	4.59 ± 0.26^b^
Epididimal adipose tissue (g)	6.45 ± 0.33	9.46 ± 0.36^a^	6.99 ± 0.66	7.10 ± 0.69	6.50 ± 0.49^b^	7.18 ± 0.96	5.76 ± 0.42^b^
Mesenteric adipose tissue (g)	5.21 ± 0.31	7.77 ± 0.24^a^	6.02 ± 0.33^b^	6.31 ± 0.42	5.90 ± 0.24^b^	6.39 ± 0.57	5.45 ± 0.39^b^
Retroperitoneal adipose tissue (g)	7.10 ± 0.75	11.34 ± 0.85^a^	8.06 ± 0.70	9.97 ± 0.76	7.24 ± 0.69^b^	9.44 ± 0.92	7.84 ± 0.79^b^

Data are presented as the mean ± SEM (n = 7). The significance of p < 0.05 is indicated by lowercase letters indicating a difference between the groups.

^a^Referring to the control group.

^b^Referring to the high-fat diet group.

^c^Referring to a diet rich in fat + ω-3 group.

^d^Referring to a high-fat diet + physical exercise group.

^e^Referring to a diet rich in fat + ω-3 + aerobic physical exercise group.

^f^Referring to a high-fat diet + chia group.

^g^Referring to high-fat diet + chia + physical exercise group. The Two-Way ANOVA test was used to compare the means. with the Tukey post-test.

### PUFA supplementation associated with aerobic training regulates food consumption, and body weight of rats fed with HFD

**Figure 1:**
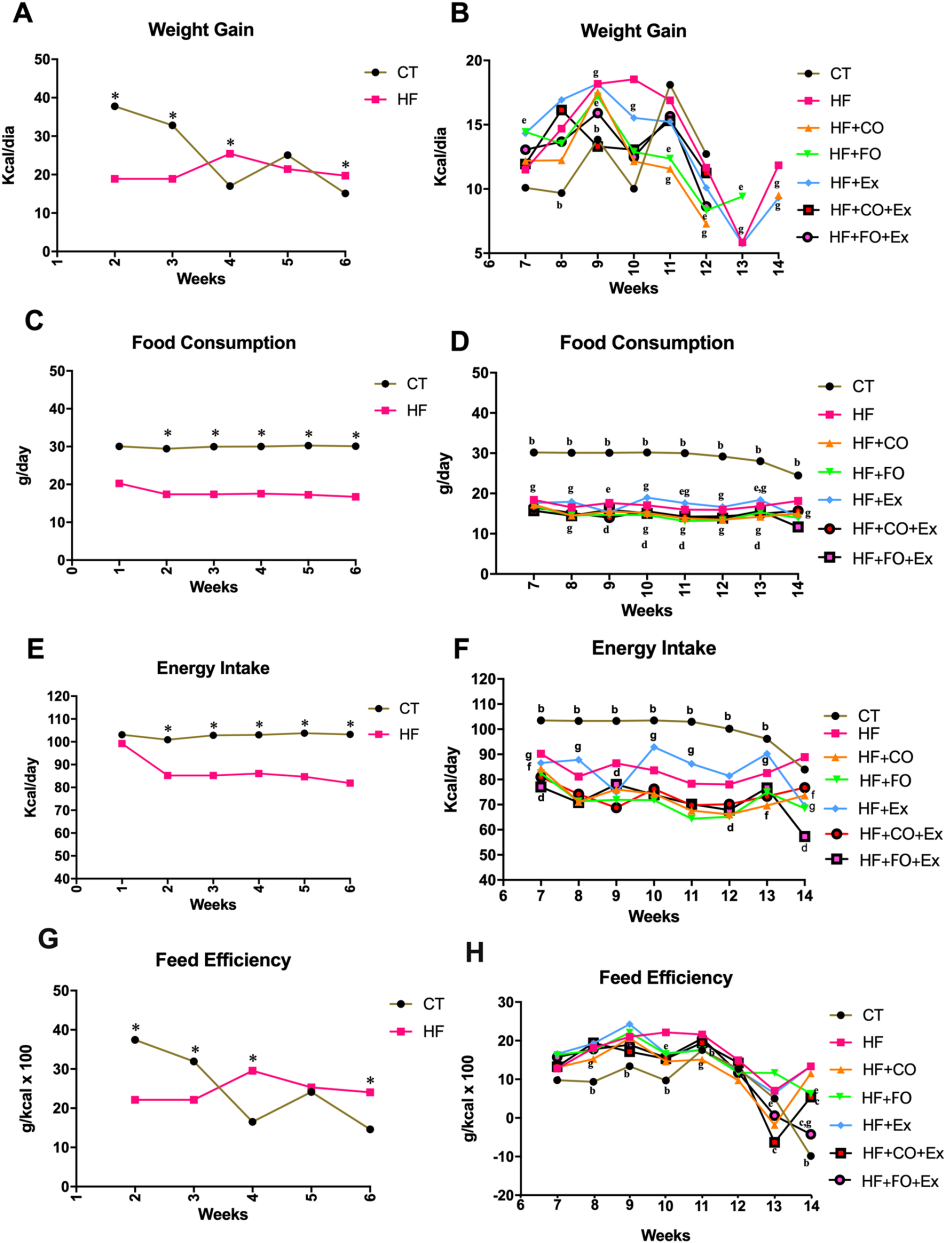
Nutritional data of weight gain, food consumption, energy intake and feed efficiency of the animals for the 14-week protocol.

During the six-week induction period, significant differences in weight gain, food consumption (p = 0.0001), and energy intake (p = 0.0001) were observed across groups. Rats in the CT groups demonstrated greater weight gain at weeks three through six (Fig. 1A), and greater food consumption and energy expenditure at weeks two through six (Fig. 1C,E) compared to the HF groups. Significant differences in weight gain (p = 0.015) and feed efficiency (p = 0.03) were also observed across groups and time, with both measures greater in the CT group at weeks two and three, but greater in the HFD groups at week four and six (Fig. 1D,M).

After the diet acclimatization period, the animals given HF were divided into six subgroups (HF, HF + Ex, HF + FO, HF + FO + Ex, HF + CO, and HF + CO + Ex) and began oil supplement and aerobic physical training until week 14. Though nominal, the groups supplemented with fish oil with and without physical exercises showed the lowest weight gain relative to all other groups (Fig. 1B). The CT group showed significantly greater food consumption than all other treatment groups (p = 0.0001; Fig. 1D) but the lowest feed efficiency of all treatments (p = 0.3; Fig. 1H). Physical exercise combined with HF did not reduce the energy intake and feed efficiency compared to the HF group (Fig. 1F,H). On the other hand, fish oil supplementation reduced energy intake compared to HF treatment (Fig. 1F,H), and physical exercise in combination with fish oil supplementation reduced feed efficiency from 10 weeks compared fish oil supplementation alone (Fig. 1H). Similarly, chia oil with and without physical exercise reduced energy intake and feed efficiency when compared to the HF + CO, HF, and HF + Ex treatments (Fig. 1F,H). Food consumption was also lower in all HFD groups compared to the CT group (Fig. 1D), however, HFD considerably increased feed efficiency and reduced energy intake throughout the experimental period.

### Effect of PUFA associated with aerobic training altering lipid profile of rats fed with HFD

**Figure 2:**
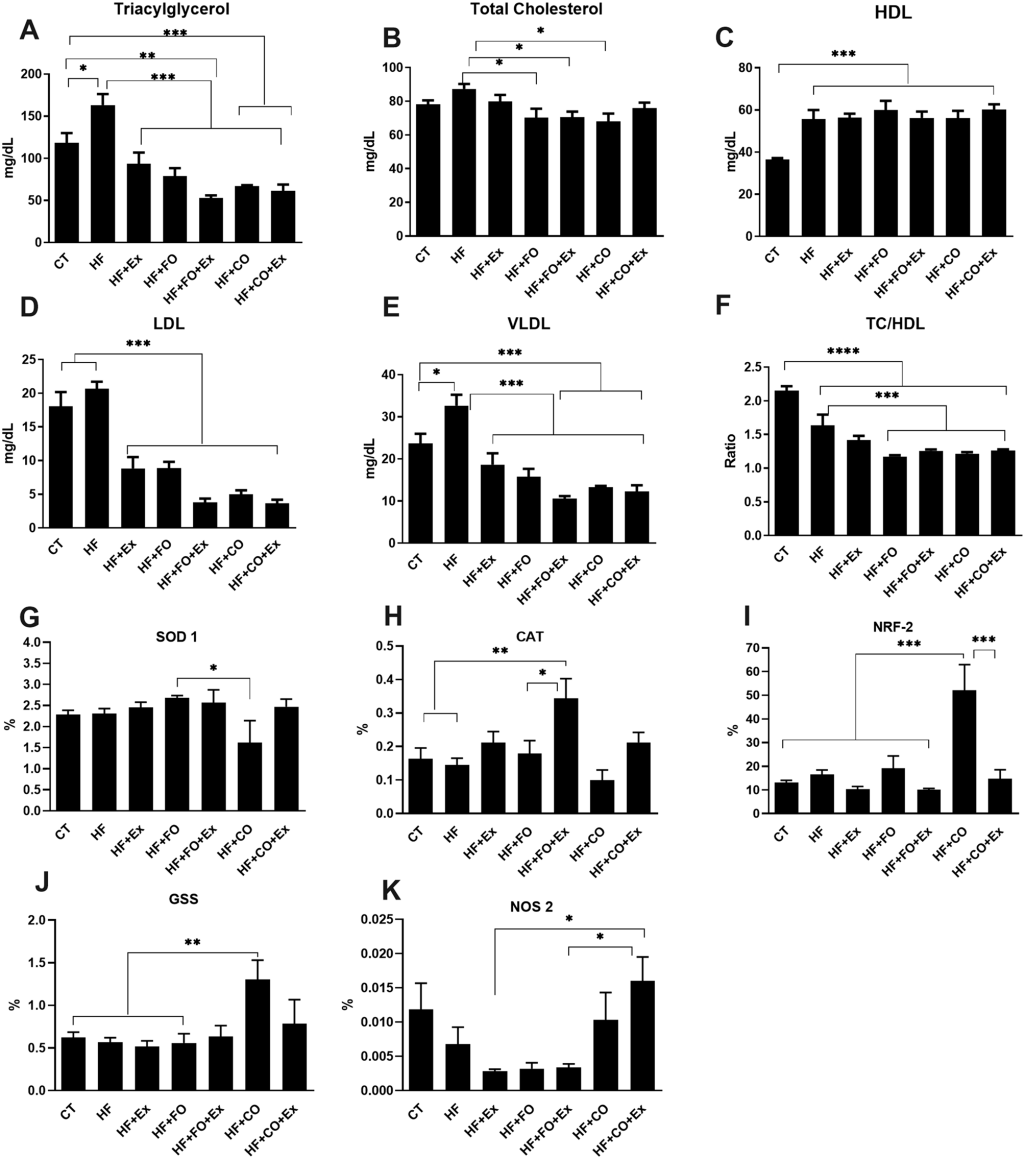
The significance of p < 0.05 is indicated by * and p > 0.05 ** indicating a difference between the groups. *CT* control, *HF* high-fat diet, *HF + Ex* high fat and aerobic exercise, *HF + FO* high fat and fish oil, *HF + FO + Ex* high fat, fish oil and aerobic exercise, *HF + CO* high fat and chia oil, *HF + CO + Ex* high fat, chia oil and aerobic exercise. (**A**) tryacilgricerol, (**B**) total cholesterol, (**C**) HDL levels, (**D**) LDL levels, (**E**) VLDL levels, (**F**) TC/HDL levels, (**G**) amount of the superoxide dismutase gene (SOD-1) in the ventral prostate; (**H**) amount of catalase (CAT) in the ventral prostate; (**I**) amount of NRF-2 in the ventral prostate; (**J**) amount of glutathione (GSS) in the ventral prostate; (**K**) amount of nitric oxide synthase 2 (NOS-2) in the ventral prostate. The Two-Way ANOVA test was used to compare the means, with the Tukey post-test.

The plasma lipid composition was analyzed the following sacrifice at 14 weeks of post-dietary initiation (Fig. 2). Comparisons across groups revealed a significant main effect of treatment on plasma TAG levels (p = 0.0001). A 2.24-fold reduction in plasma TAG was observed in the HF + FO + Ex treatment group when compared with CT (95% CI 23.63–107.7; p = 0.0003); similarly, a 3.09-fold reduction in TAG in the HF + FO + Ex group was observed relative to the HF treatment group (95% CI 68.50–152.6; p < 0.0001; Fig. 2A). In addition, TAG levels in serum from the HF + CO (95% CI 9.529–93.58; p = 0.002) and HF + CO + Ex (95% CI 14.97–99.03; p = 0.002) groups were also significantly lower than levels from the CT group (Fig. 2A). The HF group increased TAG levels when compared to CT group (95% CI − 86.90 to − 2.84; p = 0.03; Fig. 2A). Similar effects, TAG levels in serum from the HF + Ex (95% CI 27.94 to 112.0; p = 0.0001), HF + FO (95% CI 42.50 to 126.6; p < 0.0001), HF + CO (95% CI 54.40 to 138.5; p < 0.0001) and HF + CO + Ex (95% CI 59.84 to 143.9; p < 0.0001; 2.66-fold) groups were also significantly lower than levels from the HF group (Fig. 2A). The HF + FO (95% CI 0.438 to 33.62; p = 0.03), HF + FO + Ex (95% CI 0.210 to 33.39; p = 0.03), and HF + CO (95% CI 2.624 to 35.80; p = 0.03) groups exhibited statistically significant reduced in TC levels when compared to the HF group (Fig. 2B).

The main effect of treatment on serum low-density lipoprotein (LDL) was also apparent, with a 4.78-fold and 5.48-fold reduction in LDL observed following HF + FO + Ex treatment compared to CT (p < 0.0001) and HF (p < 0.0001) groups, respectively (Fig. 2D). Additionally, HF + CO and HF + CO + Ex reduce LDL levels when compared with CT (p < 0.0001) and HF (p < 0.0001) groups, respectively (Fig. 2D). The HF + Ex, HF + CO, and HF + FO statistically significantly reduced compared to CT and HF groups, respectively (p = 0.0001, Fig. 2D). The VLDL levels were also reduced in HF + FO (p < 0.0001), HF + FO + Ex (p < 0.0001), HF + CO (p < 0.0001), and HF + CO + Ex (p < 0.0001) and HF + Ex (p = 0.0001) groups when compared with HF treatment, and VLDL levels were lower in the HF + CO (p = 0.007), HF + CO + Ex (p = 0.002) and HF + FO + Ex (p = 0.003) groups compared to CT (Fig. 2E). However, VLDL levels following HF treatment increased significantly VLDL levels when compared to CT (p = 0.02; Fig. 2E).

In contrast to TAG, LDL, and VLDL levels, the CT group demonstrated significantly lower levels of HDL compared with all other groups (Fig. 2C). Nevertheless, significant changes to the ratio of TC/HDL associated with oil supplementation and aerobic physical training suggest these treatments alter plasma lipid profile (p = 0.0001) the PUFAs supplementation and PUFAs supplementation alongside exercise (HF + Ex, HF + FO, HF + FO + Ex, HF + CO, and HF + CO + Ex) groups reduced the TC/HDL ratios compared to the CT group (Fig. 2F). The TC/HDL ratio reduced significantly in the HF + FO, HF + FO + Ex, HF + CO, HF + CO + Ex groups compared to the HF group (Fig. 2F).

### Effect of PUFA diet composition associated with aerobic physical exercise altering lipid oxidative stresses of the prostate of rats fed with HFD

To investigate the effects of fish oil and chia supplementation on the balance of oxidative stress production and antioxidant capacity, we analyzed the gene expressions of SOD1, CAT, GSS, NRF-2, and NOS2 in the prostate of rats that consumed a high-fat diet (Fig. 2G–K). The expression of SOD1 and CAT mRNA was lower in the HF + CO group compared to the HF + FO group, however, the group supplemented with chia oil showed higher expression of GSS, NRF2, and NOS2 mRNA to the other groups (Fig. 2). Aerobic physical exercise increased the expression of SOD1 and CAT mRNA associated with fish oil and chia oil supplementation, however, the groups that practiced aerobic physical exercise showed low GSS and NRF2 mRNA expressions (Fig. 2). The groups supplemented with chia oil (HF + CO and HF + CO + Ex, respectively) significantly increased the expression of NOS2 mRNA when compared to the other groups (Fig. 2). In another perspective, the intervention with chia and physical exercise in the HF + CO + Ex group raised NOS 2 levels in relation to the HF, HF + Ex, and HF + FO + Ex groups (Fig. 2).

### Changes of the histopathological, mast cells and stereological analysis in the prostate of rats submitted the aerobic physical exercise and PUFFA supplementation across feeding HFD

**Figure 3:**
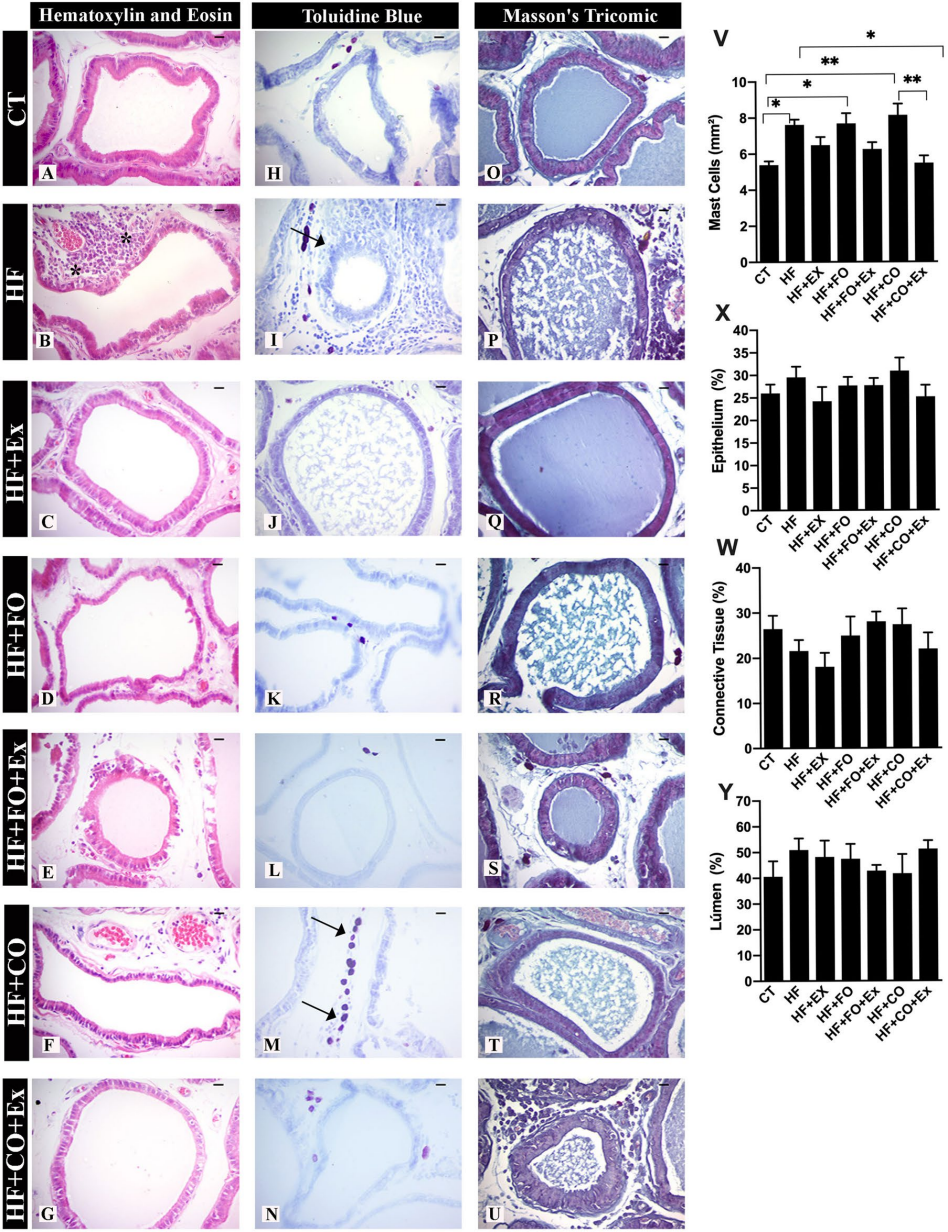
The data are presented as the mean ± SEM (n = 7). The significance of p < 0.05 is indicated by * indicating a difference between the groups. The asterisk * in H&E technique represents shows rounded acini with polymorphonuclear cells around prostate epithelium and the arrows in toluidine’s blue demonstrate the mast cells in connective tissue of prostate. *CT* control, *HF* high-fat diet, *HF + Ex* high fat and aerobic exercise, *HF + FO* high fat and fish oil, *HF + FO + Ex* high fat, fish oil and aerobic exercise, *HF + CO* high-fat diet and chia oil, *HF + CO + Ex* high fat, chia oil and exercise group. Graph V: referring to the number of mast cells per mm^2^; Graph W: referring to the prostatic epithelium; Graph X: referring to prostatic connective tissue; Graph Y: referring to the amount of prostatic lumen. Bar = 20 µm; Resolution = ×40. The Two-Way ANOVA test was used to compare the means, with the Tukey post-test.

The ventral prostate structure in the CT group presented the prevalence of acini with simple cylindrical epithelium, polarized nuclei in the basal part of the cells, and a clear supranuclear region, the latter of which corresponds to the Golgi Apparatus area (Fig. 3A). Stereological analysis show the reduction of epithelium and connective tissue in the HF + Ex group compared with groups, but without significant differences (Fig. 3X,Y), the other groups showed no differences in the stereological analysis.

It was possible to observe epithelial cell nuclei to apical areas, where they presented different heights to give a pseudo-stratified aspect to the tissue, which was frequently observed in the HF group (Fig. 3B). The presence of cells in division moving to the apical part of the epithelium indicates proliferative activity in this tissue and in some areas, the epithelium had become thick with agglomerated nuclei of various heights similar to prostatic intraepithelial neoplasia (PIN) and was showed an increase of 37% of PIN in HF group (Table 2). The proliferative inflammatory atrophy (PIA) characterized by agglomerated epithelial cells with heterogeneous phenotypes, stratified epithelial patterns, and different compacted chromatin nuclear patterns show epithelial inflammatory reactive atypia was observed in 24% and 39% of the animals of HF and HF + CO groups respectively (Table 2). Inflammatory foci observed in the animals of HF (39%) and HF + CO (24%) groups presented similar characteristics including a prevalence of lymphocytes and plasmatic cells (Table 2). The HF + FO sowed lowed alteration of how PIN and inflammation foci than compared to other groups (Table 2). On the other hand, the aerobic physical exercise showed reduced histopathological alteration in the prostate associated with supplementation or alone (Table 2). The HF (95% CI − 4.198 to − 0.2576; p = 0.01), HF + FO (95% CI − 4.264 to − 0.3236; p = 0.01) and HF + CO (95% CI − 4.720 to − 0.7796; p = 0.0023) had a higher number of mast cells in the CT group (Fig. 3V). The aerobic physical exercise reduced mast cells in HF + CO + Ex (95% CI 0.6978 to 4.638; p = 0.003) group when compared to HF + CO (Fig. 3V).

**Table 2:** Occurrence of histopathological data of experimental animals.

Groups	PIN	PIA	Inflammation
CT	17 (85)–20,76%^f,e,g^	13 (85)–14,08%^b,f,g^	13 (85)–14,28%^f^
HF	43 (114)–37,30%^e,g^	27 (114)–24,20%^e,f,g^	46 (114)–39,97%^d,f,g^
HF + Ex	16 (83)–20,30%	7 (83)–7,60%	3 (83)–3,41%
HF + FO	13 (68)–18,16%	14 (68)–21,93%	1 (68)–1,32%
HF + FO + Ex	22 (101)–21,60%^g^	20 (101)–19,42%^g^	5 (101)–5,00%
HF + CO	23 (103)–21,53%^g^	39 (103)–39,06%	26 (103)–24,69%
HF + CO + Ex	37 (100)–35,65%	22 (100)–22,01%	14 (100)–12,73%

*CT* Control Group, *HF* high-fat diet, *HF + Ex* high-fat diet and aerobic physical exercise, *HF + FO* high-fat diet and fish oil, *HF + FO + Ex* high-fat diet, fish oil and aerobic physical exercise, *HF + CO* high-fat diet and chia oil, *HF + CO + Ex* high-fat diet, chia oil and aerobic physical exercise. The results were expressed in absolute values and occurrence of percentages. The significative differences were adopted based on p < 0,05. Test Mann–Whitney.

### Effect of PUFA diet composition associated aerobic physical exercise on the modulation androgenic, lipogenic, and apoptotic prostatic

**Figure 4:**
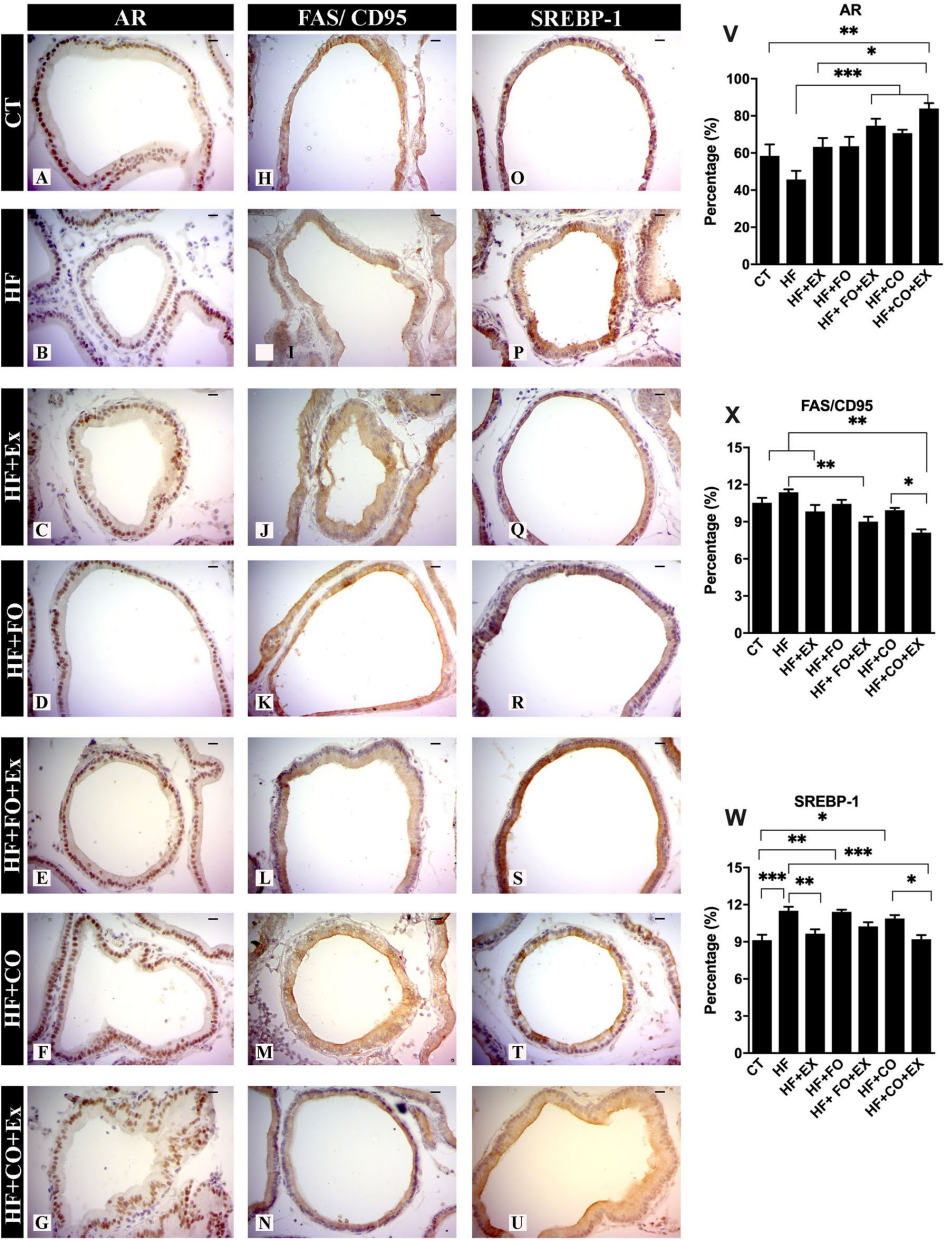
The data are presented as the mean ± SEM (n = 7). The significance of p < 0.05 is indicated by * and p > 0.05 ** indicating a difference between the groups. (**A**) CT—control; (**B**) HF—high-fat diet; (**C**) HF + Ex—high fat and aerobic exercise; (**D**) HF + FO—high fat and fish oil; (**E**) HF + FO + Ex—high fat, fish oil and aerobic exercise; (**F**) HF + CO—high fat and chia oil; (**G**) HF + CO + Ex—high fat, chia oil and aerobic exercise. Graph I: androgen receptor expression (AR); Graph II: death receptor expression (FAS/CD95); Graph III: Sterol regulatory element-binding transcription factor-1 (SREBP-1). The Two-Way ANOVA test was used to compare the means, with the Tukey post-test. Bar = 20 µm; Resolution = ×40.

We investigated the immunoreactivity of the AR, SREBP-1, IGF-1, BCL-2, BAX, and FAS/CD95 effects of fish and chia oil supplementation and physical exercise after HFD and is shown in Fig. 4. The HFD (as compared to the CT group) significantly reduced the immunoreactivity of de AR in the prostate, there was no significant difference. The HF + FO + Ex (95% CI − 47.64 to − 10.47; p = 0.0006), HF + CO (95% CI − 43.64 to − 6.474; p = 0.003) and HF + CO + Ex (95% CI − 58.01 to − 18.59; p < 0.0001) show increased AR in prostate when compared to HF group (Fig. 4A–G and V). Likewise, the mean of AR was higher in rats fed the chia oil and submitted the aerobic physical exercise (HF + CO + Ex), were significant difference in the CT (95% CI − 46.30 to − 4.745; p = 0.009) group (Fig. 4V). To compare the effects of the aerobic physical exercise and aerobic physical exercise associated with chia oil in the expression of prostatic AR it was possible to verify the difference in AR expression stimulated by chia oil in the HF + CO + Ex group when compared to the HF + Ex group (95% CI − 40.4 − 0.985; p = 0.01).

To verify the prostatic effects of the high-fat diet and the addition of fish oil and chia in the diet associated with physical exercise, we analyzed lipogenesis through the expression of SREBP-1. There was a significant increase in the SREBP-1 expression on HF (95% CI − 3.90 to − 0.83; p = 0.001), HF + FO (95% CI − 3.92 to − 0.66; p = 0.01) and HF + CO (95% CI − 3.27 to − 0.21; p = 0.01), when compared to CT group (Fig. 4W). On the other hand, aerobic physical exercise reduced the SREBP-1 expression alone (HF + Ex, 95% CI 0.39 to 3.29; p = 0.001) and associated with chia (HF + CO + Ex 95% CI 0.84 to 3.73; p = 0.001) oil when compared to the HF group (Fig. 4W). Physical exercise reduced effects lipogenic in prostate associated chia oil vs. HF + CO group (95% CI 0.22 to 3.11; p = 0.01).

**Figure 5:**
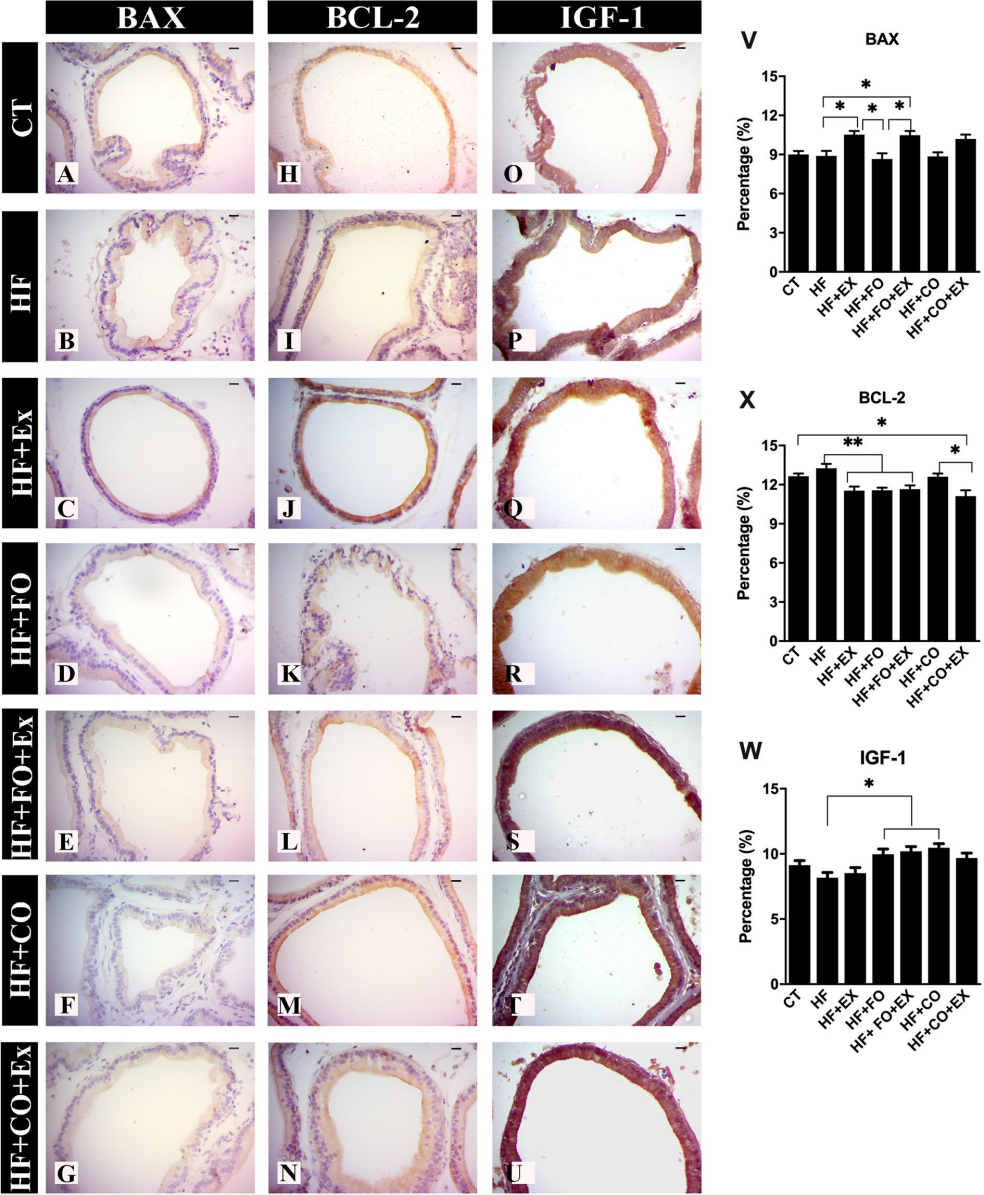
The data are presented as the mean ± SEM (n = 7). The significance of p < 0.05 is indicated by * and p > 0.05 ** indicating a difference between the groups. (**A**) CT—control; (**B**) HF—high-fat diet; (**C**) HF + Ex—high fat and aerobic exercise; (**D**) HF + FO—high fat and fish oil; (**E**) HF + FO + Ex—high fat, fish oil and aerobic exercise; (**F**) HF + CO—high fat and chia oil; (**G**) HF + CO + Ex—high fat, chia oil and aerobic exercise. Graph V: data related to BAX expression, Graph X: data related to BCL-2 expression, Graph W: data related to IGF-1 expression.

To identify the effect of the high-fat diet on epithelial progression and development of prostatic lesions and possible effects of n-3 PUFA supplementation associated with aerobic exercise, we investigated the expression of IGF-1. There was a significant increase in IGF-1 in fish and chia oil supplementation, HF + FO (95% CI − 3.43 to − 0.14; p = 0.01) and HF + CO (95% CI − 4.47 to − 0.55; p = 0.01) respectively, compared to the HF group (Fig. 5). The HF + FO + Ex increased IGF-1 compared to HF (95% CI − 3.76 to − 0.27; p = 0.05, Fig. 5).

We check the state of different proteins related to cell death and survival concerning the mitochondrial pathway after consumption of HFD and interaction of dietary n-3 PUFA and aerobic physical exercise. The HF + Ex (95% CI 0.27 to 3.14; p = 0.05), HF + FO (95% CI 0.33 to 3.09; p = 0.01) and HF + FO + Ex (95% CI 0.25 to 2.96; p = 0.05) groups showed lower BCL-2 when compared to HF group (Fig. 5). There was a more reduced level of BCL-2 in HF + CO + Ex when compared to HF + CO (95% CI 0.13 to 2.83; p = 0.05), HF (95% CI 0.78 to 3.49; p = 0.001), and CT (95% CI 0.16 to 2.87; p = 0.05) groups (Fig. 5). On the other hand, aerobic physical exercise upregulation of the BAX expression in the prostate showed in the HF + Ex group when compared with HF (95% CI − 3.19 to − 0.09; p = 0.05), HF + FO (95% CI 0.32 to 3.43; p = 0.05) and HF + CO (95% CI 0.12 to 3.23; p = 0.05, Fig. 5). The addition of fish oil to the diet associated with aerobic physical exercise, HF + FO + Ex group, significantly reduced BAX in the prostate compared to HF (95% CI − 3.14 to − 0.04; p = 0.05), HF + FO (95% CI − 3.38 to − 0.27; p = 0.05, Fig. 5).

The activity of FAS/CD95 death receptors was investigated, where the HF group showed the highest expression when compared to the HF + FO + Ex (95% CI 0.76 to 3.98; p = 0.01) and HF + CO + Ex (95% CI 1.74 to 4.79; p = 0.05) groups, respectively (Fig. 4H–N,X). Chia supplementation, HF + CO group, showed higher values of FAS/CD95 when compared to the HF + CO + Ex group (95% CI 0.28 to 3.33; p = 0.05, Fig. 4X). The HF + Ex group showed higher FAS/CD95 values when compared to the HF + CO + Ex group (95% CI 0.18 to 3.24; p = 0.05, Fig. 4X). The HF + CO + Ex group showed reduced FAS/CD95 compared to CT (95% CI 0.87 to 3.93; p = 0.001). It was evident that the stimulated pathway of apoptosis by aerobic exercise was greater expression of BAX and reduced the expression of FAS/CD95.

### Effect PUFA supplementation associated with aerobic physical exercise on inflammation after HF diet in the prostate

**Figure 6:**
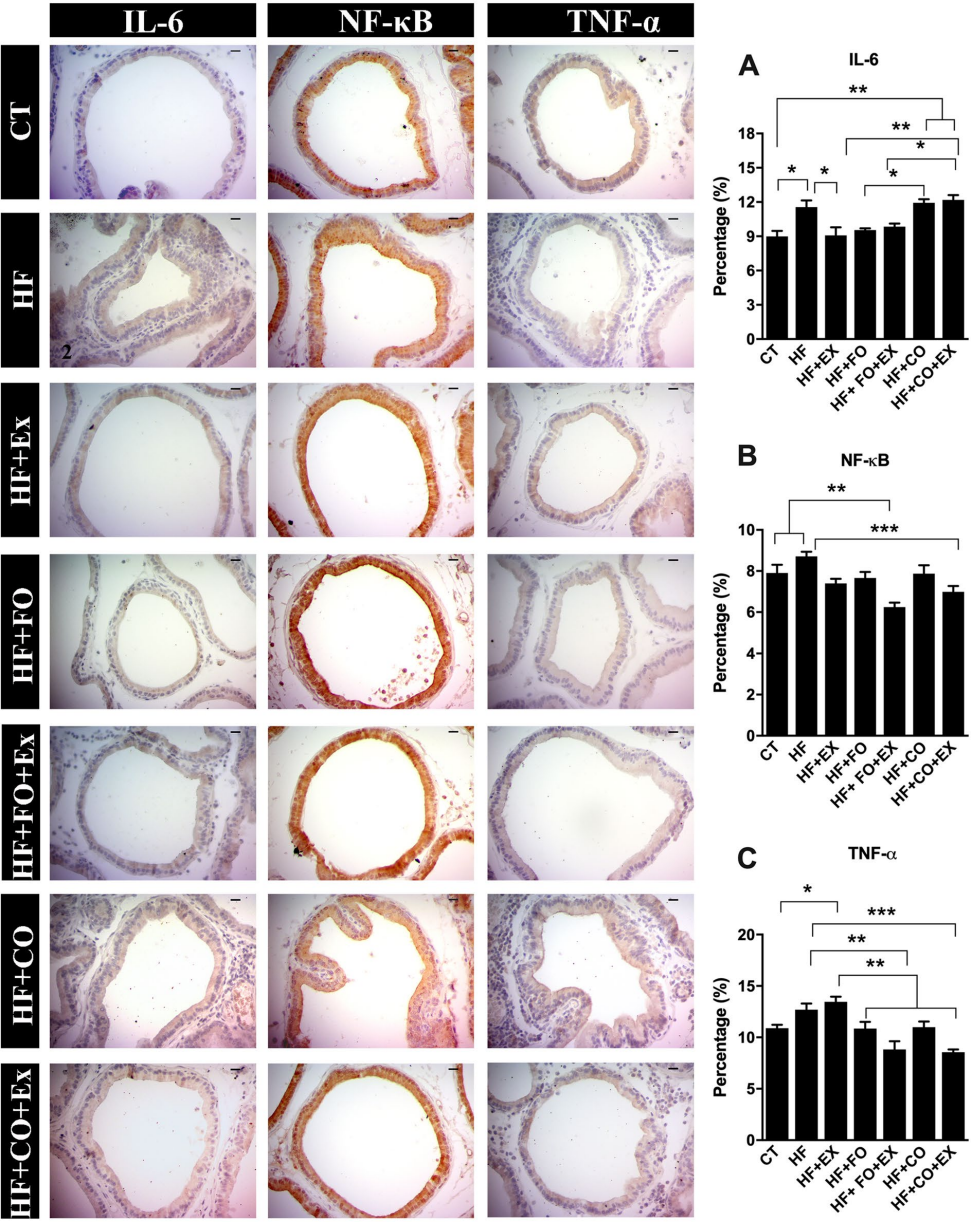
The data are presented as the mean ± SEM (n = 7). The significance of p < 0.05 is indicated by * and p > 0.05 ** indicating a difference between the groups. (**A**) CT—control; (**B**) HF—high-fat diet; (**C**) HF + Ex—high fat and aerobic exercise; (**D**) HF + FO—high fat and fish oil; (**E**) HF + FO + Ex—high fat, fish oil and aerobic exercise; (**F**) HF + CO—high fat and chia oil; (**G**) HF + CO + Ex—high fat, chia oil and aerobic exercise. Graph I: data related to IL-6 expression. Graph II: data related to NF-κB expression; Graph III: data related to TNF-α expression.

We investigated the immunoreactivity of the inflammatory markers IL-6, TNF-α, and NF-κB. The chia oil supplementation increased IL-6 (95% CI − 5.03 to − 0.84; p = 0.01) than compared to the CT group (Fig. 6). The HF + CO and HF + CO + Ex groups significantly increased IL-6 immunoreactivity compared to the HF + Ex (95% CI − 4.92 to − 0.74; p = 0.01) and HF + FO + Ex groups (95% CI − 5.19 to − 1.00; p = 0.01), respectively (Fig. 6). The HF + FO groups showed less IL-6 in the prostate compared to the HF + CO (95% CI − 4.48 to − 0.29; p = 0.05) group (Fig. 6). The association of supplementation of fish oil and aerobic physical exercise reduced IL-6 than compared to the chia oil and physical exercise group (95% CI − 4.43 to − 0.24; p = 0.05, Fig. 6).

There was a significant increase in the TNF-α immunostaining in the HF + Ex group compared to the CT group (95% CI − 5.00 to − 0.13; p = 0.05). The results showed that HF + FO + Ex (95% CI 1.27 to 6.45; p = 0.01) and HF + CO + Ex groups (95% CI 1.55 to 6.72; p = 0.001) significantly reduced TNF-α labeling compared to HF (Fig. 6). The association of the supplementation of the fisher (95% CI 2.20 to 7.07; p = 0.0001) and chia (95% CI 2.47 to 7.35; p = 0.0001) oil more physical exercise reduced TNF-α labeling in the HF + Ex (Fig. 6). Additionally, the group HF + CO + Ex was significantly reduced TNF-α when compared to HF + CO (95% CI 0.0016 to 4.87; p = 0.05). The association of fish oil supplementation with aerobic physical exercise showed a significantly reduced NF-κB when compared to HF (95% CI 0.71 to 4.10; p = 0.01) and HF + Ex (95% CI 0.19 to 3.77; p = 0.05) groups (Fig. 6). There was no significance between the other groups.

**Figure 7:**
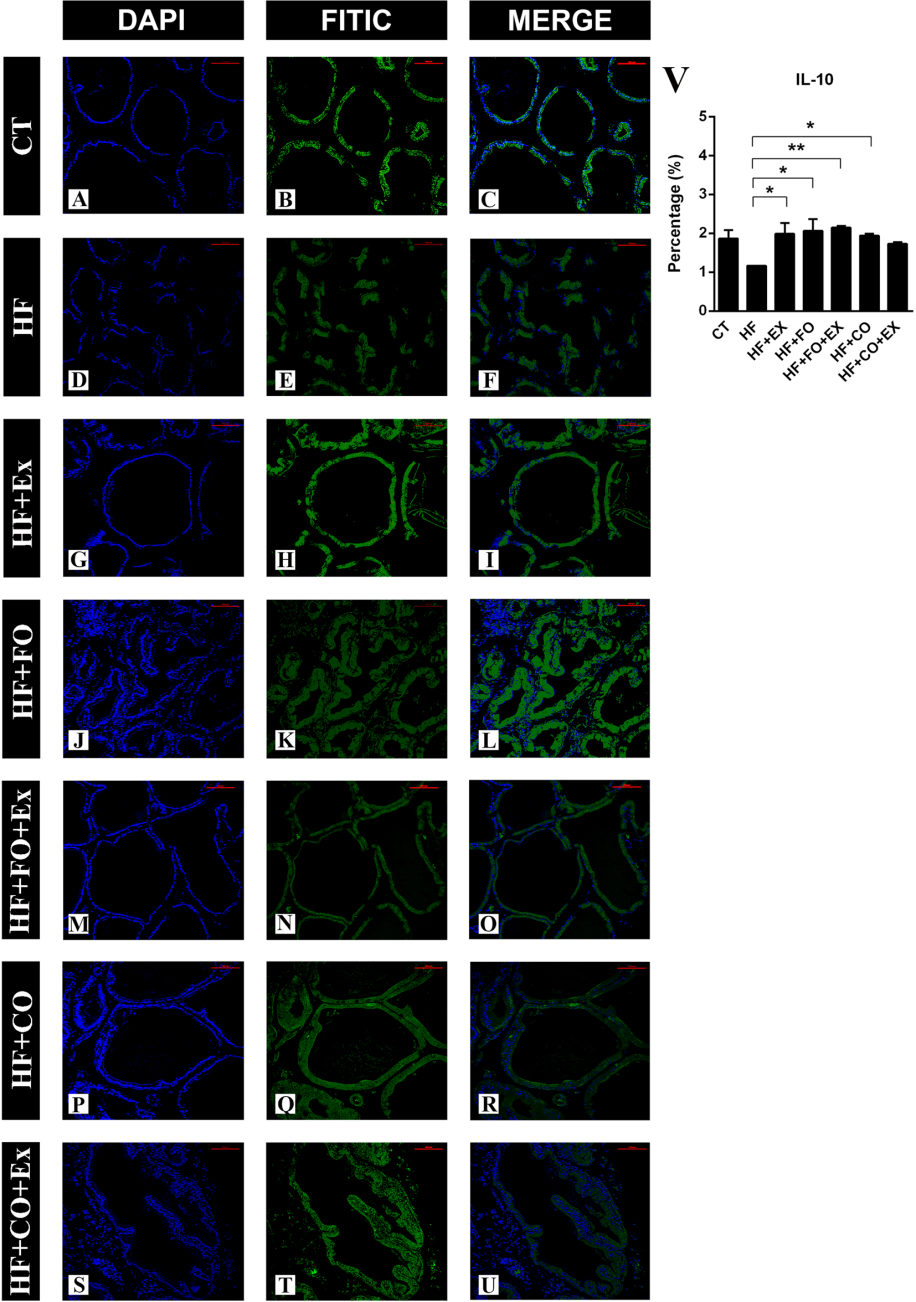
The data are presented as the mean ± SEM (n = 7). The significance of p < 0.05 is indicated by * and p > 0.05 ** indicating a difference between the groups. Immunofluorescence of IL-10 in ventral prostate of animals submitted to high-fat diet, supplemented with ω-3 PUFAs, and performed aerobic physical exercise. (**A**) CT—control; (**B**) HF—high-fat diet; (**C**) HF + Ex—high fat and aerobic exercise; (**D**) HF + FO—high fat and fish oil; (**E**) HF + FO + Ex—high fat, fish oil and aerobic exercise; (**F**) HF + CO—high fat and chia oil; (**G**) HF + CO + Ex—high fat, chia oil and aerobic exercise.

The anti-inflammatory effects of FO and CO intake associated with physical exercise in the prostate were evaluated by the expression of cytokine IL-10. We observed that the animals that were submitted to HFD showed lower expression of prostatic IL-10 when compared to the other groups. The groups supplemented with fish oil (HF + FO) showed significantly higher expression of IL-10 when compared to the HF group (Fig. 7). The groups that were submitted to physical exercise, HF + Ex, HF + FO + Ex and HF + CO + Ex, showed no difference between them, however, all were significantly different from the HF group (Fig. 7).

## Discussion

We compared the biological effects of supplementing with fish and chia oils alone or in combination with aerobic physical exercise in rats submitted to a high-fat diet. A potential mechanism by which obesity can promote severe cancer prognosis is through the induction of functional metabolic abnormalities, altering the metabolic profile, promoting inflammation and oxidative stress. The HFD exposure increased LDL-cholesterol levels, TC, and TAG, which are predominantly synthesized in the liver, are important markers of lipid metabolic disorders. Chia oil has been described as a cholesterol regulator owing to the effect of PUFA in lipid metabolism^20^. When rats were fed HFD and supplemented with fish or chia oil, HDL cholesterol levels in the plasma were not noticeably different from those not receiving oil, whereas TC levels were marginally lower relative to non-supplemented groups. Aerobic physical exercise has been reported to reduced serum levels of LDL and VLDL. Similarly, fish supplementation has been reported to reduce LDL levels^21^. The incorporation of fish and chia oils with the physical exercise clearly has more effect on lipid profile compared with fish oil supplementation or chia oil alone, as demonstrated by reduced LDL, VLDL, and TAG levels by chia oil supplementation alone. These results provide evidence that aerobic physical exercise and supplementation with fish oil may synergistically improve the amount of circulating lipoproteins and reduce lipid stocks in adipose tissue despite no apparent changes in weight during HFD consumption.

PUFAs are associated with reduced risk of several types of carcinogenesis, have been evidence in the prostate, however, this depends on numerous factors, including the source of omega-3 PUFAs. The consumption of the high-fat diet and obesity cause a reduction in testosterone and even so promote prostatic changes such as prostatitis, BHP, HGPIN^22^ until cancer^23^. A review study organized by Aucoin^4^ showed an association between increased consumption of fish oil and reduced risk of prostate cancer, however more research is needed to demonstrate the potential effects of the treatment of omega-3 and its relationship with prostate. Fish oil has higher concentrations of EPA and DHA and exceptionally, seeds of chia (Salvia Hispanic) are abundant in ALA, and the omega-3 PUFAs are considered to be activators of cholesterol esterification, an important mechanism for cholesterol reduction^24^. We associated the increase in the expression of SREBP-1 with the consumption of HFD and increased of the BHP, HGPIN in prostate independent of the AR. SREBP-1 induced prostate cancer cell proliferation, migration and invasion in vitro and promoted prostate tumor growth through the induction of FASN expression and lipid droplet formation and accumulation in prostate cells^9^. Physical exercise aerobic alone and associated with chia oil intake, a-linolenic acid (EPA and DHA precursor) reduced the levels of prostate SREBP-1 reduced PPAR activation regulated the lipogenic effects concomitant with the increase in AR. The effects of physical training promoted an increase in AR in the prostate, thus regulating the expression of SREBP-1, exhibited different efficiencies in the inhibition of proliferation.

To determine whether supplementation of chia and fish oil would cause cellular apoptosis, we checked the intrinsic and extrinsic pathways. HFD is often accompanied by decreased levels of omega-3 PUFAs^25^ and is believed to be prejudicial for the prostate. The Fas/FasL pathway is an important extrinsic apoptotic pathway and the Fas/CD95 membrane receptor initiates intracellular signaling of the apoptosis pathway by activating caspases 8 and 9. Thus, it has been shown that Fas ligand (FasL) is secreted by prostatic carcinoma cells and together with Fas/CD95 plays a key role in the development of abnormal cells^26^. Jiang^27^ reported that Fas/CD95 is more expressed in a high-grade PIN. The activation of Fas/CD95 occurs by TNF-a initiating the proteolytic cleavage pathways, in the absence of TNF-α this pathway is minimized^27^. We found that omega-3 PUFAs reduced expression of Fas/CD95 and BCL-2, and increased BAX when associated with aerobic physical exercise. It is already well documented that physical exercise promotes alteration of apoptosis in the prostate cell increases the BAX reduce proliferative ratios in the ventral prostate^28^ even in animals submitted to a high-fat diet^18^. Thus, it is possible to relate that supplementation of omega-3 PUFAs associated with aerobic exercise promotes prostatic cell apoptosis intrinsically.

Oxidative load is strongly implicated in the pathogenesis of age-related diseases, including the formation of prostate cancer tumor, and omega-3 fatty acids have antioxidant and anti-inflammatory properties, we investigated the component effects of fish oil with higher concentrations of EPA and DHA, and chia oil components with greater composition of ALA in reducing the effects of oxidative damage to DNA induced by obesity. The high-fat diet increases lipid peroxidation and higher lipid accumulation probably was related to increasing of omega-3 PUFAs with fish and chia oil supplementation. Such omega-3 is metabolized primarily at the peroxisome fraction, a well-known site of H2O2 generation, due to long-chain fatty acid structure. Therefore, the antioxidant effects of fish oil supplementation (concentration of EPA and DHA) were mainly at the mitochondrial compartment since, despite not recovering to control levels, such promoted decreased O2^·−^ directly modifying the levels of enzymatic expression of NOS2 and increased the antioxidant capacity^29^. Physical exercise is clinically associated with a reduction in lipid peroxidation by increasing the expression of antioxidant enzymes^30^. On the other hand, supplementation with chia oil increased the levels of GSS, NOS 2, and NRF-2 in the ventral prostate. Like other exogenous stimuli, chia oil can promote the NRF-2 activation pathway, to control the pro-oxidative response^31^, once activated, participates in the regulation of programmatic functions stimulated by oxidants, including autophagy, reticulum stress, and mitochondrial biogenesis.

**Figure 8:**
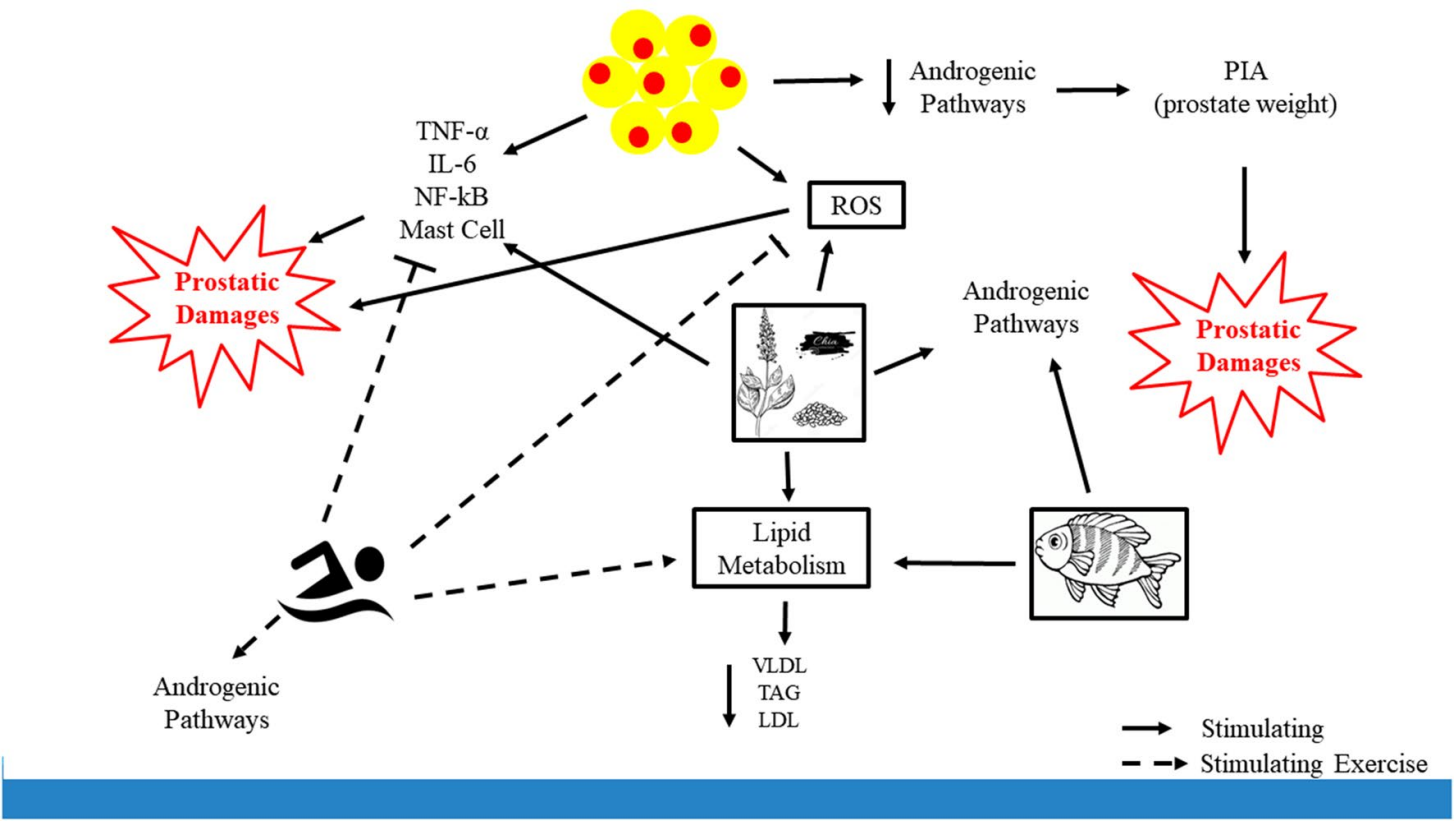
Effects of aerobic physical exercise associated with ω-3 supplementation in the prostatic obesogenic environment occurs due to an excess of high-fat diet in Wistar rats during 14 weeks of experimental protocol. The high-fat diet hypertrophy adipocytes, which, in turn, signal inflammatory cytokines such as TNF-α, IL-6, transcription factor NF-κB and mast cells recruited by the inflammatory process, cause prostatic changes. Adipocytes cause an androgenic signaling (RA), potentiating pro-oxidant factors in reactive oxygen species (ROS), which can lead to an inflammatory prostatic atrophy (PIA). In turn, aerobic physical exercise inhibits all the damage caused by the high fat diet, stimulating androgenic signaling, lipid metabolism, by reducing the levels of VLDL, TAG, LDL lipoproteins and increase as a buffering *“factor”* stimulating as anti-oxidative proteins during ROS. Finally, fish and chia oils enhance the effects initiated by physical exercise, improving androgenic signaling, lipid metabolism and ROS signaling.

Omega-3 fatty acids exhibit known inflammatory properties that suppress prostate carcinogenesis, we investigate the potential role of fish oil and chia in reducing the inflammatory effects on HFD-induced prostate epithelial cells. Statements have been published in the literature that omega-3 PUFAS are important in the quantity and quality of immune responses^32^. Fish oil intake, containing a mixture of omega-3 PUFAS, reduces the expression of IL-6, TNF-α, and NF-κB in the ventral prostate and was more efficient when associated with aerobic physical exercise (Fig. 8). NF-κB is a pro-survival nuclear transcription factor activated by a variety of stimuli, including oxidative stress. Evidence suggests that fish oil components such as DHA can attenuate the transcriptional activity of NF-κB by inhibiting translocation to the nucleus in obesity-induced prostate cells^33^. Omega-3 PUFAS -activated PPAR α can also directly interfere with the NF-κB (p50–p65 dimer) and consequently inhibit expression of the gene encoding pro-inflammatory cytokineIL-6. Possibly the higher concentration of omega-3 PUFAS in fish oil inhibited AR/NF-κB promoted down-regulation in TNF-α and COX-2, and this modification reduced PIN. Physical exercise associated with fish oil supplementation (concentration of EPA and DHA) significantly reduced prostate inflammation for increased IL-10 in the prostate. Another described effect of EPA and DHA is COX inhibition that reduces inflammation and ROS production^34^.

## Conclusions

Physical exercise and encouraging PUFA consumption are of utmost importance in the treatment of obesity and related diseases, which are characterized by variations in adipose tissue deposition, lipoprotein profiles. Employing both strategies concurrently provides additional benefits for reducing the negative effects of obesity and prostatic diseases. When we incorporate fish and chia oil supplementation into a high-fat diet, we verify the ability to prevent prostatic damage by reducing the circulating lipid profile, increasing antioxidant activity and its anti-inflammatory capabilities. This protection was more effective when associated with aerobic exercise, suggesting regulation of antioxidant activity such as higher expression of CAT and SOD-1, lower expression of IL-6, TNF-α and NF-κB as well as an increase in anti-apoptotic proteins of BAX and FAS/CD95 and reduction of BCL-2.

## Materials and methods

### Ethics statement

Experiments, all animal procedures, were conducted in accordance with the ethical principles in animal research adopted by the Brazilian College of Animal Experimentation (COBEA) and the study protocol was approved by the Ethics Committee on Animal Use (CEUA) of the Universidade do Oeste Paulista-Unoeste, Presidente Prudente (protocol number 3962).

### Animals and experimental procedures

**Figure 9:**
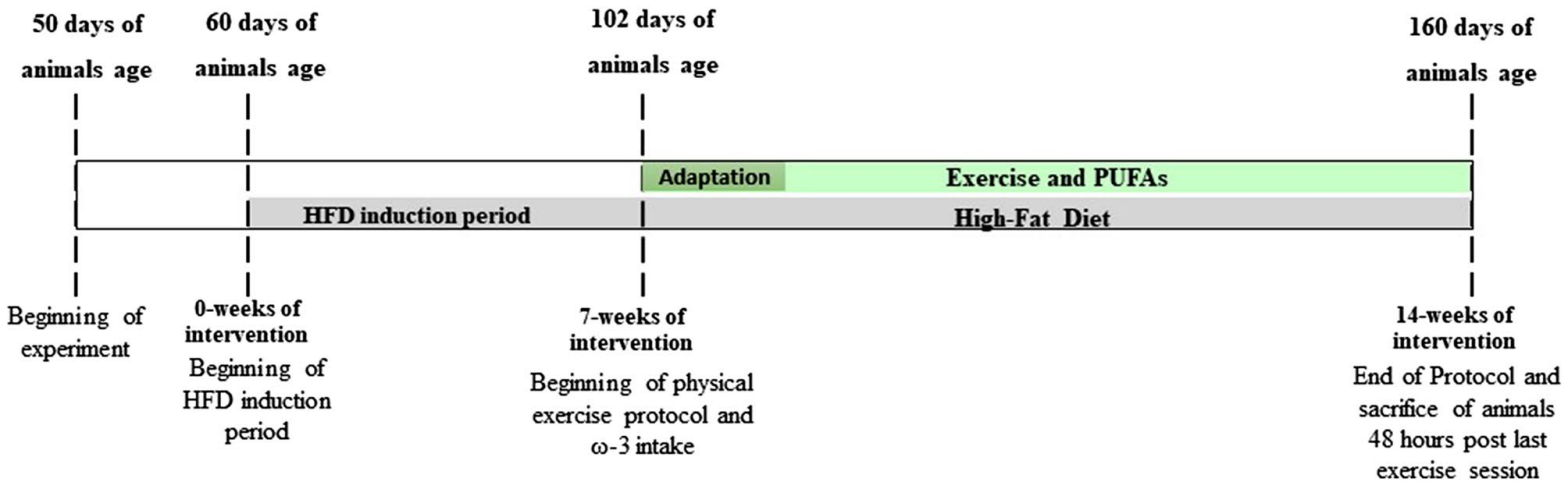
Timeline of intervention protocol for 14 weeks of a high-fat diet, fish oil supplementation, chia oil, and aerobic physical exercise.

Forty-nine adult Wistar rats (60 days old) were individually housed and maintained at 22 ± 1 °C, 60–70% humidity, and kept on a 12-h light/dark cycle for the duration of the experiment. Animals were randomized into seven treatment groups (n = 7): Adaptation phase (1st to 6th week) of the high-fat diet (HFD) was realized with all groups and control group (CT) treatment that received ad libitum standard diet and water; HFD (HF) treatment that received ad libitum high-fat diet and water; HFD with fish oil supplement (HF + FO) treatment; HFD with physical exercise (HF + Ex) treatment; HFD with fish oil supplement and physical exercise (HF + FO + Ex) treatment; HFD with chia oil supplement (HF + CO) treatment; and HFD with chia oil supplement and physical exercise (HF + CO + Ex) treatment. Beginning at 60 days old, all animals in the HFD groups underwent an HFD induction period in which they had ad libitum access to HFD, standard ration and water. At seven weeks post-adaptation (102 days old), rats began the experimental phase in which the fish oil, chia oil, and physical exercise groups began oil supplement intake and the physical exercise protocol (Fig. 9). All procedures with the animals were carried out from 1 to 6 pm.

### Dietary composition

At 60 days of age, rats were maintained on standard rat chow (commercial Supralab) or began the HFD induction period. The HFD used in this research was previously described by Estadella et al.^35^ and consisted of a hypercaloric mixture (normoproteic and HFD) containing ground and mixed commercial Supralab ration, roasted peanuts, and milk chocolate and cornstarch in a 3:2:2:2. The high-fat diet was composed by lipids (59%), carbohydrates (28%) and proteins (13%). The proximate composition of the experimental diets was evaluated according to the analytical methods recommended by the *Association of Official Analytical Chemists*. The commercial diet was composed of 24.11% of proteins, 4.27% of lipids and 52.20% of carbohydrates, while the high-fat diet was composed of 18.84% of proteins, 23.80% of lipids and 50, 4% of carbohydrates in the mixture, being 4.9 kcal/g.

### PUFAs supplementation

The PUFAs were supplemented with fish and chia oil from RSBLUMOS (RSBLUMOS Comercial Produtos Alimentícios LTDA), through gavage at 1 mL/day fish and chia oils based on prior investigation by Marinelli et al.^36^, three times a week, for 8 weeks and the animals in the other groups received the daily water gavage as a placebo so that they were submitted to the same type of stress in the animals. The fish oil was composed of saturated fatty acids (g/day) in 0.13; while chia oil was composed of 0.11, monounsaturated fatty acids (g/day) 0.20 of FO, and 0.07 in the CO. Neither oils were composed of linolenic acid and α-linolenic acid in your composition, the chia oil nor composed by 170.84 mg/day of ALA and 58.4 mg/day of linolenic acid. On the other hand, the FO was composed of 129.33 mg/day of EPA and 113.33 of DHA, and finally 3.33 mg/day of cholesterol, different from CO (Table 3).

**Table 3:** Components of chia and fish oils.

		Fish oil	Chia oil
Saturated fatty acids (g/day)		0.13	0.11
Monounsaturated fatty acids (g/day)		0.20	0.07
Polyunsaturated fatty acids (mg/day)	Linoleic (LA)	–	58.4
	Linolenic (ALA)	–	170.84
	Eicosapentaenoic (EPA)	129.33	–
	Docosahexaenoic (DHA)	113,33	–
Cholesterol (mg/day)		3.33	–
Calories oil (Kcal/1 g/day)		6	9

### Aerobic physical exercise protocol

Rats in the aerobic physical exercise groups were subjected to 30 min of swimming while wearing a weighted vest in a tank divided into sections using plastic dividers. The swimming tank was filled with water maintained at 29 °C. During the 1-week adaption phase, the rat was habituated to the swimming protocol and the vest without weights attached (Fig. 9). The training protocol was conducted three times per week. Following the adaptation phase, a weight corresponding to 3.5% of the animal's total mass was attached to the vest at the posterior region of the thorax that corresponds to a 70% exercise intensity moderate^37^. This intensity was adjusted to 70% exercise intensity at the end of the first four weeks of training in order to avoid adaptation to the protocol.

### Samples collection

At 172 days of age, 48 h after the last physical training session, after sacrifice, the animals remained overnight fasting for 12 h, had their blood collected under intraperitoneal anesthesia of ketamine (60 mg/kg) and xylazine hydrochloride (1 mg/kg), abdominal-pelvic laparotomy was performed, the ventral prostate and epididymal, retroperitoneal and mesenteric adipose tissues were removed, weighed, and processed for future analysis. The slides were analyzed and photographed in a light microscope, model AxioCam ECR5s Zeiss.

### Blood sample

Serum levels of total cholesterol (TC), high-density lipoprotein (HDL), and TAG were analyzed by blinded experimenters using a colorimetric method with Cobas C111 equipment (Roche Diagnostics-Brazil) and the ROCHE commercial kit according to the manufacturer's instructions. The TC/HDL levels were calculated based on total cholesterol (mg/dl) divided by HDL (mg/dl), the VLDL levels were calculated by TAG divided per five, the LDL was calculated using total cholesterol values minus HDL mg/dl minus VLDL ratios.

### Body weight and nutritional analyses

Body weight was also measured, to evaluate weight gain, we calculated body mass gain (Δ = final weight—starting weight). Relative prostate weight, used to evaluate the growth of prostate in different interventions, was determined as the ratio between absolute prostate weight and total animal bodyweight (g). During the experimental period, weekly consumption of water and food and changes in rat body mass were monitored. Food intake value and caloric value of ration for rodents (3 kcal/g for standard ration and 9 kcal/g for HFD) were used to obtain total energy consumption (TEI, kcal/day = average food consumption per day [g] × 3) and (ii) feed efficiency (FE, g/kcal = mean bodyweight gain/total TEI mean)^38^.

### Histologic analysis of prostate

The ventral prostate was removed, weighed, and embedded in Paraplast and the 5 µm sections were cut. These sections were then stained with hematoxylin and eosin (H&E), five histological sections by intermediate ventral prostate were evaluated and the histological fields were photographed, resulting in 10 or more fields per group. The slides were analyzed and photographed in an optical light microscope, model *AxioCam ECR5s Zeiss*. The ventral prostate was collected, fixed, prepared, and processed for histological slides, stained with hematoxylin and eosin (H&E), toluidine blue, and Masson trichrome techniques. For masson trichomic data, quantification was performed using the Weibel method^39^.

### Immunohistochemistry analysis

To block the endogenous peroxidase, the sections were subjected to a solution of hydrogen peroxide + methanol and peroxidase block. Protein blockade was performed by incubation in a blocking solution. In the next step, the sections were subjected to reaction with specific primary antibodies AR (N-20, sc-816); BAX (P-19, sc-526), BCL-2 (N-19, sc-492), NF-κB (p-65, A, sc-109); IL-6 (E-4, sc-28343); TNF-α (52B83, sc-257); SREBP-1 (H-160, sc-8984); FAS/CD95 (X-20, sc-1024), IGF-1 (G-17, sc-1422) and incubated in a humid chamber overnight. The sections were incubated with secondary antibodies, anti-rabbit HRP (IgG-HRP, sc-2030 conjugate), anti-goat antibody (IgG-HRP, sc-2354 conjugate, or m-IgGK (IgG-HRP, sc-516 conjugate) at room temperature, developed with diaminobenzidine (DAB), stained with Harris hematoxylin and with a Zeiss Axiophoto photomicroscope (Zeiss, Munich, Germany). The intensity of immunoreactivity of IL-6, NF-κB, TNF-α, SREBP-1, FAS/CD95, IGF-1, BAX e BCL-2 antigens were examined in 10 fields per animal using Image-J software version 1.50i (National Institutes of Health, Bethesda, MD, USA), and the percentage of tissue marking was quantified for each image and immunopositivity cells were used for percentage for the area. For the AR quantification, the labeling indices for each group were estimated as the percentage of stained-positive epithelial cells. The average GR index in each group was then obtained in 1,000 secretory epithelial cells per animal (from 10 fields of the intermediate region of the ventral prostate).

### Immunofluorescence analysis

To block the endogenous peroxidase, the sections were subjected to a solution of hydrogen peroxide + methanol and peroxidase block. Protein blockade was performed by incubation in a blocking solution with bovine serum albumin for 1 h diluted with PBS buffer. The sections were subjected to reaction with specific primary antibody IL-10 (NYRm, sc-73309) and incubated in a humid chamber overnight. After wash, all sections were incubated at room temperature with FITIC (goat anti-mouse (626511), Invitrogen NOVEX), and the *DAPI* was applied (DAPI:DI306). The sections were mounted with *Vectashield* (H-1000, CA94010 Burlingame, Vector Laboratories) and examined using an inverted confocal microscope. The intensity of immunoreactivity of IL-10, antigens was examined in 10 fields per animal using Image-J software version 1.50i (National Institutes of Health, Bethesda, MD, USA), and the percentage of tissue marking was quantified for each image and used for percentage for the area.

### Quantitative RT-PCR

The prostate samples were stored in the freezer at -80ºC, immersed in trizol, crushed in the tissue homogenizer, and submitted to the Trizol extraction protocol, following the protocol of the manufacturer. The concentration of the total RNA recovered was measured by spectrophotometry, all samples of total RNA were treated with DNAse before being submitted to RT-qPCR, according to the instructions of the DNAse I-Amplification Grade. Reverse transcription was performed according to the high capacity protocol using random primers as a primer oligonucleotide. The expression of NOS2, GSS, NFR2, SOD1, and CAT genes was evaluated by real-time PCR (Table 4), and for the normalization of the relative expression of the target genes, mean expression values of the GAPDH gene were used^40^. The initial standardization of real-time PCR amplification occurred on an Applied Biosystems 7500 Real-Time PCR Systems thermocycler. The calibration curve for each gene under study was made with serial dilutions of a pool of cDNA synthesized from 20 μg of prostate mRNA.

**Table 4:** Sequences of forward and reverse primers used for RT-PCR. Determined by the formula 2(-ΔCq) (Pfaffl, 2001).

Gene Primer	Forward primer	Reverse
GSS	F- TATCTCTGCCAGCTTTGGGG	95
	R- TCTTGGAAGCTTCGTTGGTCT	
NFR2	F- TCCATTCCCGAGTTACAGTGTC	91
	R- TCTCTGTCAGTGTGGCCTCT	
NOS2	F- AAACAACAGGAACCTACCAGCT	100
	R- GACCACTGAATCCTGCCGAT	
SOD1	F- GCGTCATTCACTTCGAGCAG	191
	R- CCTCTCTTCATCCGCTGGAC	
CAT	F- GCGGATTCCTGAGAGAGTGG	188
	R- GAGGGTCACGAACTGTGTCA	
GAPDH	F- CCATCACCATCTTCCAGGAG	102
	R- TCTCCATGGTGGTGAAGACA	

*GSS* Glutathione synthetase, *NRF-2* Nuclear factor erythroid 2-related factor-2, *NOS-2* Nitric Oxide Synthase-2, *SOD-1* Superoxide Dismutase, *CAT* catalase, *GAPDH* Glyceraldehyde 3‐phosphate dehydrogenase gene.

### Statistical analysis

Analysis of variance was performed for repeated measures with a 95% confidence interval adjusted by the control variables group and time. Mann–Whitney and Chi-Square statistical analysis were performed to compare the histopathological data of the ventral prostate, the analysis of variance of bidirectional estimated marginal means (ANOVA) was used to compare the mobility of the seven groups analyzed, followed by the post-test Tukey’s. For nutritional analyzes up to 6 weeks samples was used between groups a Student’s t-test for independent. All analyzes were performed using the SPSS version 25 statistical program, the p-value < 0.05 was considered statistically significant.
